# Intranasal dexmedetomidine is an effective sedative agent for electroencephalography in children

**DOI:** 10.1186/s12871-020-00978-z

**Published:** 2020-03-07

**Authors:** Hang Chen, Fei Yang, Mao Ye, Hui Liu, Jing Zhang, Qin Tian, Ruiqi Liu, Qing Yu, Shangyingying Li, Shengfen Tu

**Affiliations:** Department of Anesthesiology, Children’s Hospital of Chongqing Medical University, No.136 Zhongshan 2nd Road, Yuzhong District, Chongqing, People’s Republic of China

**Keywords:** Children, Electroencephalography, Intranasal dexmedetomidine, Sedation

## Abstract

**Background:**

Intranasal dexmedetomidine (DEX), as a novel sedation method, has been used in many clinical examinations of infants and children. However, the safety and efficacy of this method for electroencephalography (EEG) in children is limited. In this study, we performed a large-scale clinical case analysis of patients who received this sedation method. The purpose of this study was to evaluate the safety and efficacy of intranasal DEX for sedation in children during EEG.

**Methods:**

This was a retrospective study. The inclusion criteria were children who underwent EEG from October 2016 to October 2018 at the Children’s Hospital affiliated with Chongqing Medical University. All the children received 2.5 μg·kg^− 1^ of intranasal DEX for sedation during the procedure. We used the Modified Observer Assessment of Alertness/Sedation Scale (MOAA/S) and the Modified Aldrete score (MAS) to evaluate the effects of the treatment on sedation and resuscitation. The sex, age, weight, American Society of Anesthesiologists physical status (ASAPS), vital signs, sedation onset and recovery times, sedation success rate, and adverse patient events were recorded.

**Results:**

A total of 3475 cases were collected and analysed in this study. The success rate of the initial dose was 87.0% (3024/3475 cases), and the success rate of intranasal sedation rescue was 60.8% (274/451 cases). The median sedation onset time was 19 mins (IQR: 17–22 min), and the sedation recovery time was 41 mins (IQR: 36–47 min). The total incidence of adverse events was 0.95% (33/3475 cases), and no serious adverse events occurred.

**Conclusions:**

Intranasal DEX (2.5 μg·kg^− 1^) can be safely and effectively used for EEG sedation in children.

## Background

Electroencephalography (EEG) is an important tool for the clinical diagnosis of epilepsy, mental disorders, intracranial tumours and other nervous system diseases. However, for children who have difficulty falling asleep due to a lack of cooperation or anxiety before the examination, a satisfactory form of sedation can make the examination process more efficient and comfortable [1].

Many sedative drugs have been used for paediatric sedation in the past, but the use of many sedative drugs for EEG is controversial. For example, ketamine, propofol and sevoflurane can affect brain waves, which may lead to an incorrect diagnosis based on an EEG. Midazolam and chloral hydrate have been used in the past but have some shortcomings with regard to safety and sedative efficacy, respectively [2].

Dexmedetomidine (DEX) is a highly selective alpha-2 adrenergic receptor agonist that mainly acts on the alpha-2 receptor in the spinal cord and the nucleus of the locus coeruleus. DEX has little influence on haemodynamics or respiratory inhibition and has a short half-life [3, 4]. Previous studies have shown that DEX interferes little with the basic background waves of the brain; it slightly increases theta, alpha and beta activities but has no effect on the detection of an epileptic discharge [5, 6]. In addition, DEX produces a state similar to natural sleep, which can be reversed with conversation, enabling clinicians to assess a child’s cognitive status after the completion of an EEG examination [7]. As animal studies have shown, drugs can be administered through the nasal cavity, which can effectively reduce first-pass elimination, and the drug can more efficiently enter the brain through the nervous olfactory system [8]. The overall bioavailability of DEX in children with intranasal administration was reported to be 84% [9]. The use of DEX alone in paediatric sedation provides adequate sedation [10, 11]. Thus, DEX is a sedative suitable for EEG, no comprehensive studies have been performed regarding the safety and effective dose of DEX. Evaluating the safety and efficacy of 2.5 μg·kg^− 1^ intranasal DEX was the main objective of this study.

An increasing number of studies have reported the use of DEX in clinical practice. In children under deep sedation, failure to strictly meet the fasting requirement before anaesthesia did not lead to an increase in adverse events [12]. In contrast, prolonged fasting may cause anxiety in children, making them difficult to placate and leading to a reduction in sedation success rate [13].

The purpose of this study was to evaluate the safety and efficacy of intranasal DEX in paediatric EEG sedation and to provide a reference for clinical sedative use in paediatrics.

## Methods

### Patient population

This was a retrospective research study that was approved by the Ethics Committee of the Children’s Hospital affiliated with Chongqing Medical University. Our study retrospectively analysed children who underwent EEG from October 2016 to October 2018 at our hospital. Patients were sedated with 2.5 μg·kg^− 1^ intranasal DEX.

### Sedation method

The inclusion criteria for this study were children who underwent EEG in our hospital who received 2.5 μg·kg^− 1^ of intranasal DEX. Children were excluded when they met any of the following criteria: (1) A history of allergy to DEX, (2) difficult airway, (3) anatomical structural deformity of the nasal cavity, (4) severe liver or renal insufficiency and (5) severe bradycardia or atrioventricular block above II degree type 2.

Our standard sedation procedure was as follows. Children needed to fast for at least 1 h before sedation. An anaesthesiologist evaluated the patient’s general condition, history of the present illness, previous medical history, surgical history, allergy history and sedative history. Then, the anaesthesiologist created an appropriate sedative plan, and an informed consent form was signed. The child was placed in a supine position and attended by a guardian, and a nurse administered a nasal drip of 2.5 μg·kg^− 1^ DEX to the child. All the children remained lying flat for 1–2 min after the medicine was administered while we gently massaged the alae of the nose of the children to facilitate DEX absorption by the nasal mucosa. We used the Modified Observer Assessment of Alertness/Sedation Scale (MOAA/S) [14] (Table 1) to evaluate the children’s sedation state. Successful sedation was defined as an MOAA/S score less than or equal to 3 within 30 mins after the first dose of DEX. If the MOAA/S score was greater than 3 within 30 min after the first dose of DEX, an additional 1 μg·kg^− 1^ intranasal DEX was given as a “rescue” dose. If the EEG could still not be completed, inhaled sevoflurane were administered to allow the examination to be completed, which we defined as failed sedation. After drug administration, the anaesthesiologist not only assessed the child’s sedation level but also recorded heart rate (HR), pulse oxygen saturation (SpO_2_), and the occurrence of adverse events, which referred to postoperative nausea and vomiting (PONV), bradycardia, SpO_2_ reduction, etc. The EEG was performed after successful sedation, while the attending physician used a portable monitor to track the patient’s HR and SpO_2_. We defined the onset time of sedation as the time from drug administration to successful sedation. Recovery time was defined as the time from successful sedation to recovery. After the examination, the children were sent back to the sedation recovery room for further observation. Patients were discharged upon attaining a Modified Aldrete score (MAS) [15] (Table 2) of 9 or upon reaching the following states: (1) stable cardiovascular function and unobstructed respiratory tract; (2) awakened easily, with protective airway reflexes intact; (3) ability to communicate with others (age-appropriate assessment); (4) able to sit up unassisted (age-appropriate assessment); (5) for very small children or children with disabilities who were unable to exhibit the usual expected responses, a return to pre-sedation response levels or to as close to normal as possible; and (6) adequate hydration status.

**Table 1:** Modified Observer’s Assessment of Alertness/Sedation Scale

Response	Score
Agitated	6
Responds readily to name spoken in normal tone	5
Lethargic response to name spoken in normal tone	4
Responds only after name is called loudly and repeatedly	3
Responds only after mild prodding or shaking	2
Does not respond to mild prodding or shaking	1
Does not respond to a deep stimulus	0

**Table 2:** Modified Aldrete score

Criterion	Score
Activity	
Move 4 extremities voluntarily or on command	2
Move 2 extremities voluntarily or on command	1
Unable to move extremities voluntarily or on command	0
Respiration	
Able to breathe deeply, cough, and/or cry	2
Dyspnoea or limited breathing	1
Apnoeic	0
Circulation	
Blood pressure ± 20 mmHg of preanaesthetic value	2
Blood pressure ± 21 to 49 mmHg of preanaesthetic value	1
Blood pressure ± 50 mmHg of preanaesthetic value	0
Consciousness	
Fully awake	2
Arousable on calling	1
Unresponsive	0
Oxygen saturation	
Able to maintain oxygen saturation > 92% on room air	2
Needs oxygen inhalation to maintain oxygen saturation > 90%	1
Oxygen saturation < 90% even with oxygen supplementation	0

### Data collection

Sex, age, weight, American Society of Anesthesiologists physical status (ASAPS), vital signs, sedation onset and recovery times, success of sedation, adverse events, etc. were collected and recorded in Microsoft Excel 2010.

### Adverse events and handling

Adverse events were classified as severe or minor, and the occurrences of adverse events was recorded. The serious adverse events were: (1) emergency airway intervention (the use of tracheal intubation or the placement of airway aids, such as oropharynx or larynx masks); (2) laryngospasm; (3) reflux aspiration; (4) severe arrhythmia; (5) respiratory and cardiac arrest. The minor adverse reaction events were as follows: (1) bradycardia, defined as a heart rate deceleration of greater than 20% of the normal age-adjusted rate during sedation and need drug intervention (treated with atropine intravenously); (2) a significant oxygen saturation decrease, defined as an SpO_2_ of less than 90%; (3) upper respiratory tract obstruction (open airway; can be reversed with mask oxygen); (4) PONV (tilt the child’s head to one side while removing vomit from the mouth); (5) recovery delay, defined as a sedation recovery time > 2 h; and (6) rash.

### Statistical analysis

Quantitative data with a normal distribution are described with the mean ± standard deviation or median and interquartile ranges. Categorical variables are represented by a number, and the rate and 95% confidence interval (CI) were calculated. All clinical data were analysed using SPSS 17.0 for Windows (SPSS Inc., Chicago, IL, USA).

## Results

### Demographics and sedation characteristics

This study included 3475 cases of children who were examined by EEG from October 2016 to October 2018. There were 2229 (64.1%) males and 1246 (35.9%) females. The age of the children was 61.7 ± 38.9 months. The weight of the children was 19.5 ± 11.4 kg. In total, 1914 patients (55.1%) were assigned to ASAPS Class 1, 1523 patients (43.8%) were assigned to ASAPS Class 2 and 38 patients (1.1%) were assigned to ASAPS Class 3, as shown in Table 3.

**Table 3:** Demographics and sedation characteristics

Characteristics	Value
N	3475
Sex (M/F)	2229 (64.1)/1246 (35.9)
Age (months)	61.7 ± 38.9
Weight (kg)	19.5 ± 11.4
ASAPS	
1	1914 (55.1)
2	1523 (43.8)
3	38 (1.1)
4	0 (0)

Age and weight are expressed as the mean ± standard deviation; the other data are expressed as numbers (%)

### Success rate of sedation

The success rate of the initial DEX dose was 87.0% (3024/3475 cases), and the success rate of intranasal sedation rescue was 60.8% (274/451 cases).

### The time of sedation and examination

The median sedation onset time was 19 mins (IQR: 17–22 min), and the sedation recovery time was 41 mins (IQR: 36–47 min).

### Adverse events

The total rate of adverse events in this study was 0.95% (33/3475 cases). Among the adverse events, no serious adverse events occurred. Among the minor adverse events, PONV was found in 20 cases (0.58, 95% CI: 0.3–0.8%); SpO_2_ was reduced in 6 cases (0.17, 95% CI: 0–0.3%); upper respiratory tract obstruction was observed in 3 cases (0.09, 95% CI: 0–0. 2%); rash was observed in 2 cases (0.06, 95% CI: 0–0.1%); heart rate decreased by more than 20% of the normal age-adjustment and drug intervention was required in 1 case (0.03, 95% CI: 0–0.1%); and recovery delay occurred in 1 case (0.03, 95% CI: 0–0.1%), as shown in Table 4.

**Table 4:** Adverse events

Adverse events	N (%)	95% CI
PONV	20 (0.58)	0.30–0.80
Significant SpO_2_ reduction	6 (0.17)	0–0.30
Upper airway obstruction	3 (0.09)	0–0.20
Rash	2 (0.06)	0–0.10
Bradycardia requiring drug intervention	1 (0.03)	0–0.10
Delayed awakening	1 (0.03)	0–0.10

## Discussion

In the past, many sedative drugs were used clinically for moderate and deep sedation during paediatric examinations, but EEG examination is unique. Many sedative drugs that act on the central nervous system interfere with brain waves. Some studies have shown that ketamine can selectively inhibit the thalamic neocortex system and activate the medulla oblongata and limbic system as well as indirectly excite the brain waves and increase the theta wave due to the “separation anaesthesia” effect of ketamine [16]. A small dose of propofol can increase beta waves, and a high dose of propofol increases the delta wave frequency due to the double action of propofol at different dosages [17]. Sevoflurane affects the number of slow delta and alpha waves [18]. The sedative drugs midazolam and chloral hydrate were used in the past; while they exhibited little interference with EEG, they had a long half-life, many side effects, high sedation failure rates and other undesirable characteristics [19, 20]. DEX has a good sedative effect when it is administered via intranasal administration or intravenous injection [21]. Additionally, DEX has been widely used for sedation before paediatric examinations [22]. Because DEX is a new sedative, its use in EEG procedures is limited. We summarized the experience of intranasal DEX in EEG, which can provide a reference for clinical practice.

In our study, the success rate of the initial dose of 2.5 μg·kg^− 1^ DEX was 87.0% (3024/3475 cases). A recent study have found that 90% of the effective dose of intranasal DEX sedation was 3.28 μg·kg^− 1^ in children [23]. Another study found that the 50% effective dose and the 95% effective dose of intranasal DEX increased with increasing age in patients under 3 years of age [24]. The success rate of sedation in our study was slightly lower than that in previous studies [11]. We believe the reason for this observation is that the age (61.7 ± 38.9 months) of the children in this study was higher than that in previous studies. Therefore, we speculate that when older children are sedated, the initial dose can be appropriately increased to improve the sedation success rate, but further research is needed to verify the safety and efficacy of this approach. Notably, Jenny Bua found DEX is an attractive and reliable sedative in preterm neonates undergoing MRI. We also hope to further study the safety of DEX in preterm neonates during EEG [25].

In this study, no serious adverse events occurred in 3475 paediatric cases. In contrast to previous studies, there were no respiratory-related severe adverse events (such as laryngospasm and bronchospasm) while using intranasal DEX [26]. This result again confirms the safety of this sedation method.

The most common adverse event was PONV (0.58%), which resolves on its own after rest. Through the monitoring of vital signs after sedation, we found that DEX can slow the heart rate of children [27]. Therefore, we speculate that because of the specific physiology of children, the heart rate slowed down, and the symptoms of nausea and vomiting appeared. The incidence of PONV was higher in our study than in previous studies on the use of intranasal DEX for various paediatric examinations [26]. This observation may be due to the various nervous system diseases in patients who underwent EEG in our study.

There are still some limitations to our research. First, the subjects in this study were children aged from half a month to 204 months (61.7 ± 38.9 months), and there are differences in the physiological characteristics among different age groups. In addition, the anaesthetist evaluated the sedation depth of the children with external stimulation after nasal sedation, but this monitoring was not continuous, so there was a certain error while recording the onset time and recovery time of sedation; the recorded time was often longer than the actual time. Additionally, this was a retrospective study. Continuous blood pressure monitoring was not performed routinely as standard practice in hospital clinics, so we cannot report whether the children had hyper or hypotension as a possible side effect during the whole examination process, which also needs to be confirmed by prospective studies.

## Conclusion

An intranasal DEX dose of 2.5 μg·kg^− 1^ for paediatric EEG examinations has a high sedation success rate, quick recovery and low incidence of adverse reactions.

## Data Availability

All data generated or analyzed during this study are included in this published article. Raw data are available upon reasonable request from the corresponding author.

## Abbreviations

ASAPS: American Society of Anesthesiologists physical status
DEX: Dexmedetomidine
EEG: Electroencephalography
HR: Heart rate
IQR: Interquartile ranges
kg: Kilogram
MAS: Modified Aldrete Score
mg: Milligram
min: Minute
ml: Millilitre
MOAA/S: Modified Observer’s Assessment of Alertness and Sedation
PONV: Postoperative nausea and vomiting
SpO_2_: Pulse oxygen saturation
μg: Microgram
